# News and Notes

**Published:** 1903-11

**Authors:** 


### NEWS AND NOTES.

A SEVERE epidemic of typhus is reported in the interior of China. Ten thousand persons have died.

FOUR hundred cases of typhus are reported in two regiments of the Bavarian infantry at Hammelburg, Bavaria.

TWO wards of the Grady Hospital were recently destroyed by fire. There were no fatalities. The origin of the fire was not ascertained.

DR. ARCHIBALD N. SINCLAIR has been appointed a member of the Board of Medical Examiners of the territory of Hawaii, in place of Dr. C. L. Garvin, who has resigned.

SURGEON-GENERAL RIXEY has decided to urge on Congress the enlargement of the General Naval Hospital at Portsmouth, Va. The institution now has 200 beds, and he will recommend that it be enlarged so as to accommodate 500.

PROFESSOR GUIDO BACCELLI, the distinguished physician of Rome, who at present holds the portfolio of agriculture in the Italian government, has been elected a corresponding member of the Paris Académie des Sciences in the section of medicine and surgery in the room of the late Professor Ollier, of Lyons.

THE president of the American Congress on Tuberculosis to be held in Washington, D. C., April 4, 5 and 6, 1905, announces Alfred Meyer, of New York city, consulting physician to the Bedford Sanitarium for Consumptives, to be chairman of a committee in charge of the section on sanitarium treatment of tuberculosis. It is probable that the climatic and other methods of treatment will be comprised under the work of this committee.

---

THERE is said to be a very curious and interesting class of epileptic cases noticed among the invalided soldiers who have returned from South Africa. The number of these cases observed in South Africa was astounding—over 2,000, according to the report. The epileptic attacks were not caused by wounds, but by harsh, screaming detonations of modern firearms, particularly cannons, and by the explosions of lyddite shells. It is feared the epileptic attacks will be incurable.—*Medical Times.*

---

NEWSPAPERS state that Benjamin Hamrick, of Webster Springs, W. Va., and his nine sons beat the record for remarkable families. At a recent family reunion it was found that the aggregate height of the ten male members of the Hamrick family was 62 feet 3¾ inches, while the combined weight aggregated 1,741 pounds. The shortest man was 6 feet 1 inch, and the tallest man 6 feet 5¾ inches; the lightest was the father, who weighed 155 pounds, while the heaviest was 226 pounds.—*Exchange.*

---

DR. GEORGE A. SOPER, the expert appointed from the State Board of Health, has been investigating the subject of typhoid fever in Ithaca since March last. He states that there are now in the city nine cases, of which two are of recent origin, and five cases under suspicion. Cases have been attributed to an outbreak in a summer home above the in-take of the city water-supply from Six Mile creek. Nine-tenths of the citizens of Ithaca wish to have an artesian water-supply, but a powerful minority is backing what is considered to be an inadequate filtration plan.—*New York Medical Journal.*

BOARD OF MEDICAL VISITORS OF SCHOOLS.—All the members of the Board of Medical Visitors for the Public Schools of Atlanta who served during the last year were reelected at a meeting of the Board of Education which was held yesterday afternoon at the Boys' High School. Dr. L. P. Stephens, president of this board, has called a meeting of the members for this evening at 8 o'clock in the Boys' High School, and a full attendance is desired. The plans of D. W. Winburn for the two new schools to be erected soon will be discussed in regard to sanitation, ventilation and light, and other important business will be brought before the board. The Board of Medical Visitors is as follows: Calhoun — Drs. A. W. Calhoun, M. Hull and S. Barnett. Ivy—Drs. A. G. Hobbs, M. Hoke and E. B. Block. Walker—Drs. W. C. Robinson, J. G. Earnest and A. H. Baskin. Davis—Drs. Roy, W. B. Parks and J. N. Brawner. Marietta—Drs. E. C. Davis, V. O. Hardon and W. J. Asher. Williams—Drs. J. S. Todd, A. W. Stirling and J. R. Garner. State—Drs. J. L. McDaniel, L. M. Crichton and W. M. Cawhern. Boulevard and Edgewood—Drs. R. R. Kime, C. J. Vaughan and C. E. Murphy. Crew—Drs. L. Amster and Floyd McRae. Girls' High School—Drs. W. S. Kendrick, J. E. Summerfield and E. Van Goidtsnoven. Formwalt—Drs. C. G. Giddings and S. A. Visanska. West End—Drs. T. D. Longino, W. A. Crowe and W. Jay Bell. Ira—Drs. J. P. Rosser, Dunbar Roy and L. B. Clarke. Boys' High School—Drs. L. P. Stephens, J. M. Crawford and W. S. Goldsmith. Bell and Night School—Drs. Bernard Wolff, T. H. Sims and L. C. Fischer. Fair—Drs. J. C. Olmstead, W. C. Warren and J. Gaston. Fraser—Drs. T. V. Hubbard, George Brown and C. E. Boynton.

